# *Active Women over 50* online information and support to promote physical activity behaviour change: study protocol for a pilot trial

**DOI:** 10.1186/s40814-020-00627-9

**Published:** 2020-06-27

**Authors:** Geraldine Wallbank, Catherine Sherrington, Leanne Hassett, Dominika Kwasnicka, Josephine Y. Chau, Fiona Martin, Philayrath Phongsavan, Anne Grunseit, Colleen Canning, Marian Baird, Roberta Shepherd, Anne Tiedemann

**Affiliations:** 1 Institute for Musculoskeletal Health, School of Public Health, Faculty of Medicine and Health, The University of Sydney and Sydney Local Health District, PO Box M179, Missenden Road, Camperdown, 2050 Australia; 2 Discipline of Physiotherapy, Sydney School of Health Sciences, Faculty of Medicine and Health, The University of Sydney, PO Box 170, Lidcombe, NSW 1825 Australia; 3 Faculty of Health Sciences, School of Public Health, Curtin University, GPO Box U1987, Perth, WA 6845 Australia; 4 SWPS University of Social Sciences and Humanities, Aleksandra Ostrowskiego 30b, 50-505 Wrocław, Poland; 5 Melbourne School of Population and Global Health, University of Melbourne, 333 Exhibition Street, Melbourne, 3000 Australia; 6 Department of Health Systems and Population, Macquarie University, 75 Talavera Road, North Ryde, NSW 2109 Australia; 7 Charles Perkins Centre, Sydney School of Public Health, The University of Sydney, Camperdown, NSW 2006 Australia; 8 Department of Media and Communications, Faculty of Arts and Social Science, The University of Sydney, Camperdown, NSW 2006 Australia; 9 Discipline of Work and Organisational Studies, Sydney Business School, The University of Sydney, Camperdown, NSW 2006 Australia

**Keywords:** Physical activity, Exercise, eHealth, Website, Health coaching, Feasibility, Behaviour change, Study protocol, Pilot trial

## Abstract

**Background:**

Physical activity has many physical and mental health benefits and can delay the development of disability in older age. However, uptake of this health behaviour is sub-optimal in women in their middle and older age. This trial aims to establish the acceptability and feasibility of the *Active Women over 50* programme involving online information, telephone health coaching and email or SMS support to promote physical activity behaviour change among women aged 50 years and over.

**Methods:**

Sixty community-dwelling women who are insufficiently active according to national guidelines, will be recruited and randomised to 1) receive the *Active Women over 50* programme or 2) a wait-list control. *Active Women over 50* is a 3-month physical activity programme guided by behaviour change science, providing access to a website, one telephone-delivered health coaching session from a physiotherapist and 8 email or 24 SMS messages. The primary outcome is the proportion of participants at 3 months post-randomisation who would recommend participation in the programme to another person like themselves. Secondary outcomes are feasibility measures: rates of recruitment, retention, completeness of outcome data and uptake of telephone support; and intervention impact measures: accelerometer-assessed average steps/day, proportion of participants meeting national guidelines on moderate to vigorous physical activity; and questionnaire-assessed quality of life, exercise perceptions, mood, physical functioning and self-reported physical activity. Intervention participants will also complete a follow-up survey to assess impressions of the intervention and adoption of strategies for physical activity participation. Data will be analysed descriptively to guide the design of a larger trial. Between-group differences in secondary outcomes will be used to estimate effect sizes for sample size calculations for a fully powered randomised controlled trial.

**Discussion:**

This feasibility pilot trial of an efficient eHealth and health coaching intervention guided by user input and behaviour change theory, will inform future interventions to address low physical activity participation among an under-active group at risk of future disability.

**Trial registration:**

ANZCTR, ACTRN12619000490178, registered 26 March 2019

## Background

Physical inactivity is a significant but modifiable public health problem. Globally, it is associated with 5.3 million deaths [1] and has an economic cost of INT$67.5 billion per year [2]. Physical inactivity is as important a risk factor for developing chronic disease and disability as smoking and obesity [3].

There is compelling evidence of the benefits of physical activity for physical and mental health at all ages [3] and maintenance of independence in older age [4]. Regular physical activity can prevent or help manage some health conditions and has been reported to delay disability by up to 15 years in older women [4]. The national guidelines for physical activity recommend Australian adults to accumulate 150 to 300 min of moderate-intensity or 75 to 150 min of vigorous-intensity physical activity, or an equivalent combination of both moderate and vigorous activities each week, and to do muscle-strengthening activities on at least 2 days each week [5]. Achieving the national guidelines for physical activity in middle age has longevity benefits regardless of baseline physical activity [6].

Physical activity participation is sub-optimal in women in their middle age and very low in older women. Sixty-five per cent of women over the age of 55 years and 80% of women over the age of 75 years do not participate in sufficient regular physical activity to gain health benefits [7]. While some women become more physically active with retirement [8] this may be too late to prevent disability in older age. By 2023, Australian women will be retiring at an older age with access to the senior pension age increasing to 67 years [9]. Commencement of sufficient regular physical activity before retirement therefore needs to be a priority and maintained in the long term to promote independent and healthy ageing.

Barriers to physical activity participation for women are unique. The 2014 national survey showed that women have higher sedentary time and greater carer responsibilities than men [10, 11]. These barriers, coupled with the demands of paid work and low confidence for participating in physical activity in the absence of a previous habit, place women in their middle-age years particularly at risk of being insufficiently physically active to maintain good health. This population would benefit from a targeted and supported evidence-based approach to increase their physical activity in a way which is achievable and sustainable.

Health psychology uses a variety of tools and behaviour change techniques that can be made available to middle-aged women to support them to increase their levels of physical activity and to maintain these levels in the long term. A theoretical basis incorporating the self-determination theory (SDT) [12] and Behaviour Change Wheel and COM-B system model of behaviour [13] shed light on key factors necessary for the initiation and maintenance of physical activity [14]. SDT considers an individual’s motivation behind the choices they make by addressing competence, autonomy and psychological relatedness, and the COM-B system model of behaviour considers an individual’s capability, opportunity and motivation to achieve behaviour change. Key factors of SDT and COM-B include plentiful resources (psychological and physical), effective self-regulation, maintained motivation to be active, positive habits and positive environmental and social influences. Effective behaviour change interventions often include techniques that tap into these behavioural factors. For instance, resources in the form of health information and programme information provides an individual with physical capability and competence; goal setting, self-monitoring, action and coping planning support self-regulation and opportunities for behaviour change; relatable peer role models stimulate motivation; using habit formation techniques help to develop automaticity forming positive responses to contextual cues; and supporting development of positive habits, i.e. increasing physical activity and reducing sedentary behaviour [15–17].

Behavioural interventions can be delivered digitally, via web and smartphone, in order to increase scalability for population-level impact [18]. eHealth online interventions using web, email and SMS-based platforms have a number of advantages over face-to-face interventions, especially where they can be delivered to mobile phones: they have wide reach, comparatively low cost of implementation and delivery, and flexibility of intervention use at times and location convenient for the user [19, 20]. Mobile phone access to websites, video, email and short message service (SMS) provides a portable, everyday use context for receiving eHealth messages, promoting higher engagement and adherence [21, 22]. There is emerging evidence supporting the use of SMS to promote health behaviour [23], and including email reminders to prompt participants’ health behaviour change [24]. One disadvantage of online interventions is potential disengagement as people prefer the personalised nature of face-to-face interactions. To address this, effective interventions often include phone or video sessions with a health coach who guides the participants, provides social support and tailors behaviour change techniques to participants’ abilities and preferences, supporting participants’ autonomy, in line with SDT. A review of health coaching has shown a beneficial effect on physical activity [25], so including a skilled health coach with an online intervention is likely to increase engagement and consequently the effectiveness of the intervention. To date, there are limited programmes that flexibly support middle-aged women in becoming more active and staying active for life which can be implemented at scale. Online behavioural interventions augmented by health coaching provide a potential scalable solution to the current problem of inactivity among this age group.

This trial aims to establish the acceptability and feasibility of the *Active Women over 50* programme, a 3-month programme comprising online information, telephone health coaching and follow-up email or SMS support to promote physical activity behaviour change among women aged 50 years and over.

## Methods/design

### Trial design

**Fig. 1:**
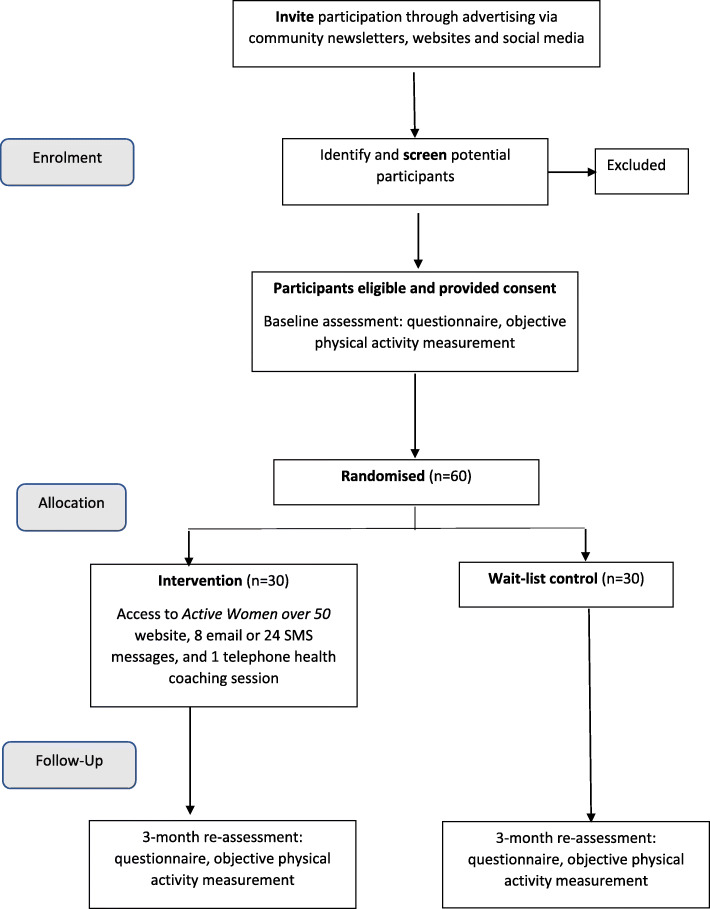
CONSORT flow diagram for the pilot and feasibility study *Active Women over 50*

We will conduct a feasibility pilot study using a randomised controlled trial (RCT) with wait-list control. The CONsolidated Standards Of Reporting Trials (CONSORT) flow diagram is illustrated in Fig. 1. This trial has been designed according to the CONSORT statement [26] and will be reported according to the Standard Protocol Items: Recommendations for Interventional Trials (SPIRIT) statement [27], and with reference to the Template for Intervention Description and Replication (TIDieR) checklist [28] in Table 1.

**Table 1:** Intervention description using the Template for Intervention Description and Replication (TIDieR) checklist [28] for *Active Women Over 50* study

1. Brief name	*Active Women over 50* online physical activity information and support
2. Why	Physical activity has many physical and mental health benefits, including the delay of developing disability in older age. Yet the uptake of this health behaviour is particularly low in older women and sub-optimal in working women in their middle age. While commencement of regular sufficient physical activity in middle-age years needs to be a priority for healthy ageing, women over 50 years have unique barriers to becoming more active (e.g. higher sedentary time, greater carer responsibilities, work demands). Behavioural interventions delivered remotely and digitally via web, email and SMS platforms offer wide reach and provide flexibility of intervention use at times and location convenient for the user. Telephone health coaching can also be delivered remotely and provides tailoring of the intervention to a person’s abilities and preferences. A theoretical basis incorporating the Behaviour Change Wheel and COM-B system model of behaviour, Social Determination Theory has been used to design the intervention and choice of behaviour change techniques. The intervention targets barriers unique to women over 50 and will incorporate the behaviour change techniques including goal setting, problem-solving, action and coping planning, review of behavioural goals, self-monitoring, social support and social comparison, information provision, persuasion about capability and habit formation to facilitate and sustain behavioural change.
3. What materials	Participants will have access to the internet. The website will be accessed by the internet and will provide 1) information for the benefits of physical activity including links specific to women, different health conditions and ages; 2) resources and strategies for how to become physically active including a pre-exercise screening tool and exercise intensity guidelines [29], guide on getting started, internet-based programme resources such as the *Active and Healthy* website [30], *parkrun* website promoting free, weekly community walk/runs [31], links to sporting groups and suggestions of smartphone applications; 3) inspirational stories of people becoming physically active including professionally produced video case study interviews of four real-life female women aged over 50 who have overcome barriers to increase their physical activity; and 4) the opportunity for participants to share their own ideas and inspirational stories via the website. Participants will also receive their choice of either 8 email messages or 24 SMS messages embedded with behaviour change techniques that will link back to and reinforce the website content and provide further motivation to increase their physical activity. Participants will also receive one telephone health coaching session with a trained research physiotherapist trained in physical activity behaviour change techniques.
4. What procedures	Access to the intervention website will be provided to participants upon randomisation to the intervention group. Participants will be asked not to share the website to avoid contamination. Participants will also be given the choice to receive either 8 emails or 24 SMS messages over the 3-month study period, and a mutually agreed time will be made for the health coach to contact the participants within 2 weeks of receiving access to the intervention. The health coach will document behaviour change techniques used in the telephone session. All participants will have email access to the trial manager for any further enquiries.
5. Who provided	The study manager will provide participants with access to the website, regular messages and liaise appointment times with the health coach. Health coaching will be provided by a tertiary-trained physiotherapist employed by the study with research experience delivering telephone-based health coaching. The coach will have completed courses through Wellness Coaching Australia [32], HealthChange Australia [33] and Medicoach [34] in motivational interviewing and behavioural intervention techniques.
6. How	Participants will be notified by email upon randomisation to the intervention group and will gain access to the intervention website via a web address. Frequency of accessing the website will be at the discretion of the participant. The email will also give participants the choice to receive regular messages via email or SMS and will ask for available times when the health coach may contact them. The health coach will be provided with participants’ available times, contact, demographic and current physical activity details by the study manager.
7. Where	Participants will access the intervention via the internet and telephone at a location of their convenience.
8. When and how much	After participants are given the intervention website address, they have access to the website at any frequency they choose. The regular email or SMS messages link back to the website to support behaviour change with the reinforcement of information and suggested strategies. The health coaching telephone call will be made within 2 weeks of accessing the intervention. There will be no cost to participate.
9. Tailoring	All participants will receive the same online resource (website and email or SMS messaging content), but the opportunity to talk with a health coach will allow for tailoring to individual’s preferences, needs and circumstances so that physical activity can be adopted and maintained. Participants will be advised to seek individual advice from a health professional if they are concerned about commencing physical activity or have an injury.

### Eligibility criteria

Women are eligible for inclusion in the trial if they are aged 50 years and over, and community-dwelling residents in New South Wales, Australia. Potential participants will be excluded if they have limited English language skills, do not have access to the internet, have a medical condition that precludes participation in regular physical activity or are already sufficiently active in accordance with Australian physical activity guidelines [5].

### Recruitment

Participants will be recruited through advertising in newsletters and websites of community organisations, university and local health districts, via the study recruitment website, www.activewomenover50.org.au, word-of-mouth and social media. Participation is cost-free and no incentives are offered for participation. Potential participants will be screened for eligibility via an online survey. Recruitment commenced 3 May 2019, and at the time of submission of this manuscript, 66 participants had been recruited, 56 randomised and 0 participants had completed follow-up measures.

### Randomisation

Following confirmation of eligibility and completion of informed written consent and the baseline measures, participants will be randomly allocated by a research assistant to the intervention or control group in equal numbers using computer-generated randomisation. Allocation will be determined using concealed allocation, via a randomisation schedule embedded in a secure web-based software platform, *REDCap* (Research Electronic Data Capture) [35, 36] hosted at The University of Sydney, Australia. A variable block randomisation schedule will be prepared from a computer-generated list of random numbers by a researcher not involved in participant recruitment.

### Intervention group

The intervention is described by the TIDiER checklist (in Table 1). Intervention components and choice of behaviour change techniques were informed and underpinned through integration of the Behaviour Change Wheel and COM-B system model of behaviour [13], SDT [12] and user input from a convenience sample of women in the target population. Examples of intervention functions and behaviour change techniques (BCTs) used in the *Active Women over 50* intervention are shown in Table 2. The *Active Women over 50* intervention was developed following our previous trial [38] investigating an information and support intervention with a 3-month follow-up period to enhance physical activity in university and health service-employed women over 50 years of age. The feedback from participants indicated a need for more support (manuscript under preparation). The intervention group will receive access to the *Active Women over 50* website plus one telephone consultation with a research physiotherapist trained in health coaching, plus either 8 email or 24 SMS follow-up motivational messages over a 3 month period.

**Table 2:** Examples from the *Active Women over 50* intervention coded within the COM-B domains, intervention functions and behaviour change techniques (BCTs)

COM-B domains	Intervention function	BCTs	Examples
Psychological capability	Education	Information about health consequences (5.1)	**Website:** “Why be Active?” section: “Regular physical activity can make you feel good and improve your self-esteem and confidence creating opportunities to socialise and meet new people”
			**SMS/Email:** Week 5: “Strength and balance exercises can help to prevent falls. You can do these exercises while watching TV. Look under “Tips” at www.[study website name].com/getting-started”
			**Health coaching:** Verbal education about physical activity for falls prevention
	Training	Instruction of how to perform a behaviour (4.1)	**Website:** “How to be Active-Getting started-Tips & hints” section: Video links, e.g. falls prevention exercises
			**SMS/Email:** Week 6: “Is something blocking your activity plans? Think of likely solutions. Perhaps break down goals into easier steps. Or ask an exercise professional for advice.”
			**Health coaching:** Instruction on balance exercises where appropriate
		Graded Tasks (8.7)	**Website:** “How to be Active-Getting started-7 steps to getting started” section: “Start small and gradually build up the amount of time you are active, or the intensity you can be active, or your goals. Use this pre-exercise questionnaire [link], and find out what light, moderate and vigorous exercise intensity is for you.”
			**SMS/Email:** Week 1: “Every bit of exercise counts! Start small and gradually build up.”
			**Health coaching:** Advice about gradual increase in physical activity
	Enablement	Goal setting (behaviour and outcome) (1.1, 1.3)	**Website:** “How to be Active-7 Steps for Getting Started-Step 4” and “How to be Active-Tools to keep going” section: refers participants to scheduling and goal setting resources.
			**SMS/Email:** Week 2: “Work towards your activity goals! Write down your when-where-how-action plan for the week. Put your plan & goals on your fridge, or where you can see them.”
			**Health coaching:** Advice on setting Specific, Measurable, Achievable, Realistic, Timely (SMART) goals, setting goals with the participant.
		Action planning (1.4)	**Website:** “How to be Active-Tools to keep going” section: provision of physical activity weekly planner and physical activity charting templates
			**SMS/Email:** Week 1: “How do others keep motivated to be active? Many find making a plan with firm goals helps. Have a look at www.[study website name]/tools-to-keep-going”
			**Health coaching:** Advice on action planning, referring to the website for physical activity weekly planner and charting templates
		Self-monitoring of behaviour (2.3)	**Website:** “How to be Active-Tools to keep going” section: provision of templates to chart physical activity “How to be Active-Mobile apps” section: suggestions of mobile apps to assist self-monitoring
			**SMS/Email:** Week 3: “Hi [FirstName], Track your activity on a calendar, chart or phone app so you can see your progress. See www.[study website name].com/mobile-apps for app suggestions.”
			**Health coaching:** Refer participant to website physical activity planning and charting templates; discussion/suggestions around wearables and phone apps for tracking activity.
		Problem-solving (1.2)	**Website:** “How to be Active-Frequently Asked Questions (FAQ)” section: responds to FAQs about how to be active. E.g. “I’ve never really been active before. Where do I start?” and “What do I do it I think physical activity is boring?” and “I have longstanding aches and pain. Is physical activity safe?”
			**SMS/Email:** Week 3: “If lack of time stops you from being active, how can you fit activity into your day? Replace some TV or device time with activity? Or have a walking meeting?”
			**Health coaching:** Identify motivators and barriers to physical activity, advice on action planning
Reflective motivation	Persuasion	Feedback on behaviour (2.2)	**SMS/Email:** Week 6: “It’s 6 weeks since you committed to getting active. Congratulations on your efforts so far!”
		Verbal persuasion about capability (15.1)	**Website:** “How to be Active-Getting started” section: “For you, being more physically active may be simply a matter of spending more time doing the things you already enjoy doing such as taking the dog for a walk or gardening. Or maybe it's doing an activity with someone else. Pick something to do that you enjoy - then you're more likely to stick at it.”
			**SMS/Email:** Week 11: “Things can get in the way of you keeping up your activity. What strategies have you learnt to deal with difficult situations? Do you want to share these? www.[study website name]/contact”
			**Health coaching:** Motivational interviewing to increase self-efficacy
		Social comparison (6.2)	**Website:** “Be Inspired” section: video case studies produced for the website of real women and their experiences with physical activity – why they do it and what keeps them going; weblinks to articles from social media; opportunity for participants to share their own physical activity story via the website.
			**SMS/Email:** Week 1: “How do others keep motivated to be active?... Have a look at www.[study website name].com/tools-to-keep-going.”
			**Health coaching:** Provision of examples of what other women 50+ have done to increase physical activity when appropriate
		Credible source (9.1)	**Website:** Investigator institution logos on website “Be Inspired” section: Video case studies from women aged 50+ “How to be Active-Find an activity or sport” section: links to larger reputable organisations, e.g. NSW Health, parkrun Australia
			**SMS/Email:** Investigator contact details on each email footer, links to website and larger reputable organisations, e.g. NSW Health, parkrun Australia
			**Health coaching:** Study health coach will be a Physiotherapist trained in behavioural intervention techniques and health coaching; participants referred to study health coach by study manager
	Modelling	Demonstration of the behaviour (6.1)	**Website:** “Be Inspired” section: Video links, e.g. This Girl Can, Females in Football
			**Health coaching:** Set goals with the participant; send links to balance exercise videos
Automatic motivation		Habit formation (8.3)	**Website:** “Be Inspired” section: “Penny” video case study talking about importance of routine; “How to be Active-7 steps for Getting started-Step 2” section: “Find out locations, times, costs of the activity or sport.” Location and times will then act as cues to action. “How to be Active-Tools to keep going” section: provision of physical activity weekly planner and physical activity charting templates
			**SMS/Email:** Week 7: “Activities that easily fit into your daily life are much more likely to become a habit. Why not put a note on the fridge, or set a phone reminder to be active?”
			**Health coaching:** Identify motivators for developing healthy habits
Social opportunity	Enablement	Social support (unspecified) 3.1	**Website:** “Be Inspired-Your story” section: “Share your physical activity story and read others’” to share experiences of physical activity
			**SMS/Email:** Week 4: “You're more likely to succeed if you tell someone your plans to be active - a relative, friend, or even your GP.”
			**Health coaching:** Identify social supports to support physical activity participation
Physical opportunity	Enablement	Adding objects to the environment (12.5)	**Website:** Links on website for finding physical activity opportunities
			**SMS/Email:** Week 3: “See www. [study website name]/find-an-activity-or-sport to pick an activity you may enjoy. While being active you could listen to music, a podcast or invite a friend.”
	Environmental restructuring	Prompts/cues (7.1)	**Website:** “How to be Active-Tools to keep going” section: provision of goal setting, physical activity planner, physical activity charting templates participants can print out and put up. “Be Inspired-Penny’s story” section: recommendations about the routine of putting out clothes at night as prompt to go to gym in the morning
			**SMS/Email:** Week 7: “Activities that easily fit into your daily life are much more likely to become a habit. Why not put a note on the fridge, or set a phone reminder to be active?”
			**Health coaching:** Provide suggested prompts/cues to activity, e.g. stick goals/action plans on fridge

Note: In brackets BCT numbers in line with [37]

**Fig. 2:**
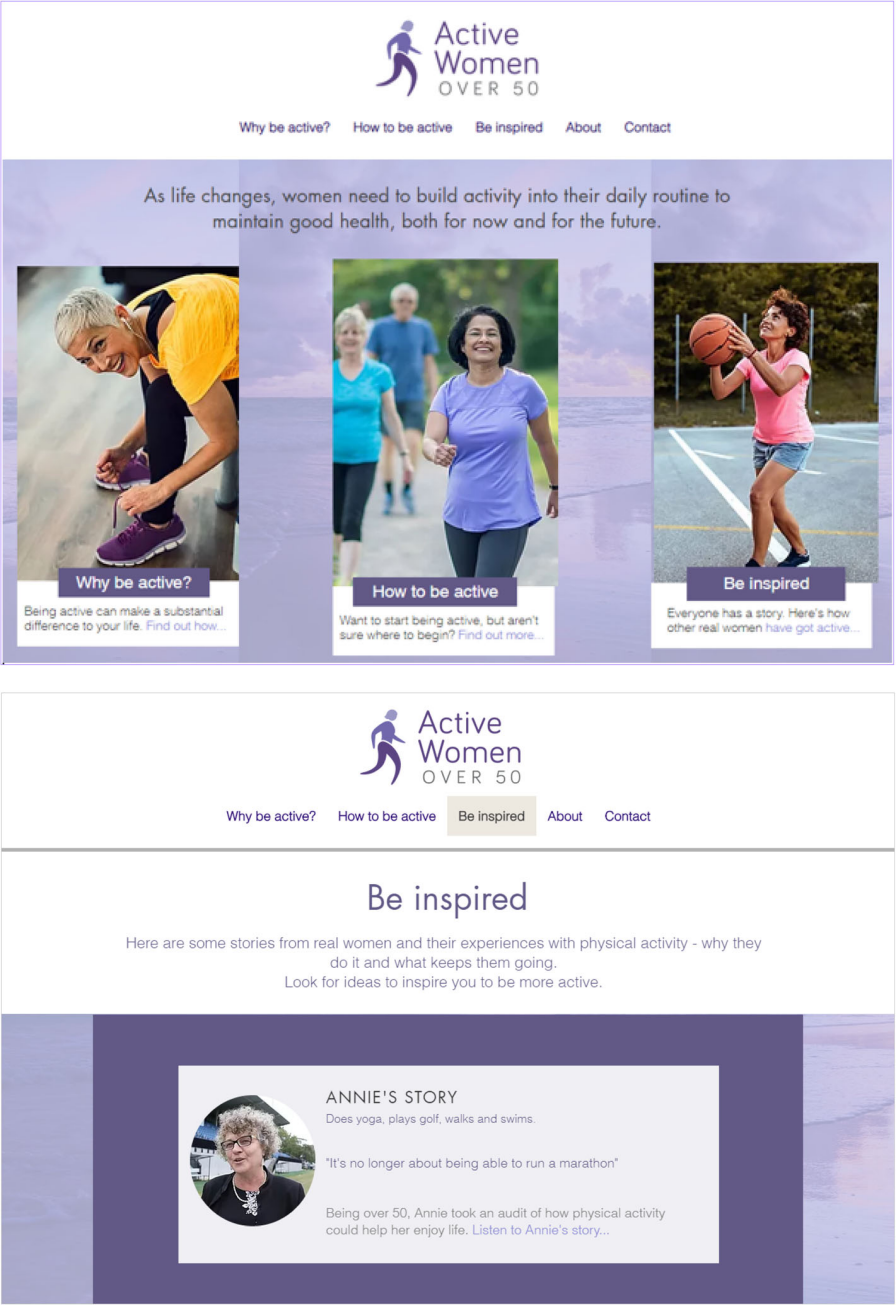
Intervention screenshot examples—main subpages. Note: main subpages of the website intervention include (a) *Why be Active*, capability and motivation; (b) *How to be Active*, opportunities and skills; (c) *Be inspired*, coping role models; (d) information about the programme; and (e) contact details for enquiries

Intervention participants will receive access to the *Active Women over 50* website (Fig. 2) via a web address and will be asked not to share the website to avoid study contamination. The website will deliver content via three main webpages namely, “Why be Active?”, “How to be Active” and “Be Inspired”. The “Why be active?” webpage will emphasise the importance of becoming active from middle age for maintenance of health and physical function and prevention of falls in older age. The *Active Women over 50* website content will include evidence-based information about the impact of even small increases in physical activity on health and longevity. The “How to be active” webpage will include practical suggestions on becoming more active and guidance on the setting of SMART (Specific, Measurable, Achievable, Realistic and Time-related) goals and self-assessment of barriers to physical activity participation and solution generation as key behaviour change techniques. The website will link to external sources of support/information, such as the NSW Ministry of Health-funded *Get Healthy* free health coaching service [39] and the *Active and Healthy* online directory [30] of physical activity opportunities. The website will also provide suggestions of resources and internet applications supporting behaviour change and habit formation, and other links for information about physical activity for different health conditions and services available. The “Be inspired” webpage will include inspirational video case studies of “success stories” of four “real-life” women aged over 50 who have managed to increase their physical activity levels in the face of mobility, health or practical difficulties and the benefits that they have experienced. To stimulate motivation and provide an interactive element to the website, there will also be the opportunity for participants to share their own ideas and inspirational stories via the website. Frequency of use of the *Active Women over 50* website will be at the discretion of the participant.

Participants will receive one telephone health coaching consultation, which will occur within 2 weeks of randomisation. The consultation session will facilitate goal setting and physical activity behaviour change, using evidence-based theoretically informed behaviour change principles. Health coaching will be provided by a tertiary-trained physiotherapist with additional training in motivational interviewing and behavioural intervention techniques, and experience in delivering telephone-based health coaching in the context of research studies.

Participants will receive either 8 email messages or 24 SMS messages depending on their preference. The messages will be similar in content and aim to support physical activity using behaviour change techniques, informed by the COM-B system model of behaviour [13] and SDT [12]. Behaviour change techniques will include providing practical tips, addressing barriers, providing motivation by social modelling and reminders of the benefits of physical activity, assisting with action planning, problem-solving and time management. These messages will also contain a link back to the *Active Women over 50* website.

### Control group

Participants randomised to the control group will be waitlisted to receive access to the *Active Women over 50* full programme after the 3-month follow-up data collection period.

### Outcomes

#### Primary outcomes

The primary outcome will be participant acceptability of the intervention and study methods. A global measure will assess whether participants would recommend participation in the study to someone else like themselves. Participants will be asked to select “yes” or “no/unsure” to the question “Would you recommend the *Active Women over 50* study to another person such as yourself?” at 3 months post-randomisation.

#### Secondary outcomes

Secondary outcomes will measure the feasibility of the study methods and intervention and the impact of the intervention. Feasibility will be evaluated with process indicators at 3 months post-randomisation using study records to measure the 1) rates of recruitment assessed by the proportion of people screened who met the inclusion criteria and proportion of eligible people who agreed to participate in the study; 2) representation of sociodemographic characteristics of participants screened and consenting to participate in the study; 3) retention of participants assessed by the number of participants who withdraw consent or participation; 4) mode by which people heard about the study; 5) completeness of outcome data assessed by accelerometer and survey questionnaire outcome data; 6) uptake of telephone health coaching consultation with the research physiotherapist, assessed by the proportion of intervention participants who make an appointment with the research physiotherapist health coach; 7) uptake of the *Active Women over 50* website resource, assessed by the proportion of intervention participants who used the website, the website content that was accessed and the intensity of website usage, measured by Google Analytics; and 8) engagement with the email messages, assessed by the proportion of intervention participants who open the automated email messages sent by secure email marketing website *Vision6* [40]. Records will be kept when different aspects of the intervention are offered, delivered and received by each participant.

Intervention impact will be measured via a questionnaire and *Actigraph GT3X+* accelerometer, as detailed below, at 3 months post-randomisation assessing 1) the average number of steps taken per day (accelerometer measured), 2) the proportion of participants taking part in at least 150 min of moderate-intensity physical activity per week or at least 75 min of vigorous-intensity physical activity per week (as recommended by the Australian Physical Activity Guidelines) [5] (accelerometer measured), 3) exercise perceptions as measured by the *Exercise Benefits and Barriers Scale* [41], 4) mood as measured with the positive and negative subscales of the *Positive and Negative Affect Schedule* [42], 5) self-reported physical activity as measured by the *International Physical Activity Questionnaire* [43], 6) physical functioning as measured by the function component of the *Late Life Function and Disability Instrument* [44], and 7) health-related quality of life as measured by the *EQ-5D-5 L* survey [45].

### Data collection procedure

Both the questionnaire-based measures and accelerometer-measured physical activity will be completed at baseline and repeated at 3 months post-randomisation. Data will be assessed or extracted by a research assistant blinded to group allocation. Questionnaires will take approximately 30 min to complete and will be accessed via an online survey link (*REDCap* [36]) or sent in hardcopy by post, as preferred by the participant.

The intervention group will complete an additional questionnaire at 3 months post-randomisation to assess their impressions of the intervention, perceived benefits and barriers, adverse events, usage of goal setting and their physical activity plans following the intervention. The questionnaire will ask both open- and closed-ended questions, for example “Did you set yourself a physical activity-based goal during the study?” and “What, if anything, helped you to achieve your goals?” An email or phone call will be sent to any participants with incomplete outcome measures to improve data completeness and participant retention.

### Objectively measured physical activity

*Actigraph GT3X+* is able to accurately estimate how physically active a person is throughout the day by measuring 3D body accelerations and is a valid instrument that has been extensively researched in the physical activity and public health field [46]. Participants will wear the *Actigraph (GT3X+)* for 7 consecutive days during waking hours (except during water-based activities or bathing). The accelerometer will be posted to participants with instructions for usage, along with an activity calendar to complete over the 7-day period and a pre-paid envelope for returning the device to the research team. Activity counts will be collected at a sampling frequency of 30 Hz and reintegrated to 60-s epochs for data analysis. Accelerometer data will be manually checked against participant activity calendars to verify wear time, and erroneous data will be excluded prior to analysis.

### Data management

To ensure participant confidentiality, the final dataset will contain re-identifiable information only. All study data will be entered onto a password-protected database and maintained on a firewall-protected local network server at The University of Sydney. Paper files will be stored in a locked filing cabinet in the Chief Investigator’s office. The database and paper files will only be accessed by study staff. All publications associated with the results of the study will involve de-identified data, so participant confidentiality will be maintained.

### Analysis of outcomes

Feasibility outcomes will be analysed descriptively to guide the design of a larger trial. Accelerometer data will be analysed using *ActiLife 6* software with time spent in different activity intensity levels calculated using the Troiano 2008 cut-points [47]. Acceptable wear time will be defined as at least 4 days with 10 h or more per day of valid wear time. Periods of 90 min or more of consecutive zeros (indicating non-use) will be considered as non-wear time.

Odds ratios will be calculated to assess the acceptability and feasibility of the study on the dichotomously scored primary outcome (the likelihood a participant would recommend participation in the study to someone else such as themselves) to assess the effect of group allocation on the dichotomously scored secondary outcome (proportion of people achieving physical activity in accordance with national guidelines) [5], adjusting for baseline scores. The study methods will be considered feasible if at least 80% of all participants complete follow-up measurements, or 80% of intervention participants access the intervention website or participate in the telephone health coaching session. Intervention versus control group differences in secondary outcomes, analysed with general linear models adjusting for baseline scores, will estimate effect sizes for sample size calculations for a larger trial and will provide an indication of likely intervention effect. This will be the progression criteria for proceeding to a fully powered randomised controlled trial.

A *p* value of < 0.05 will be considered statistically significant. Analyses will be pre-planned, conducted while masked to group allocation and will use an intention-to-treat approach. Analyses will be conducted using the *Stata 14* software package.

### Sample size justification

There will be 60 community-dwelling females aged 50 years and over recruited for the pilot trial [48], allocated in equal numbers to the intervention and control groups. This sample size will provide feasibility data and estimates for sample size calculations for planning a future fully powered trial [49].

### Ethics and dissemination

The trial protocol has been approved by the Human Research Ethics Committee at The University of Sydney, Australia (approval number 2019/075). The trial was prospectively registered (ACTRN12619000490178, 26 March 2019). Results will be disseminated via peer-reviewed journal articles and international conferences, and a lay summary will be made available to all participants at the completion of the study.

## Discussion

Active ageing is at the core of this highly innovative project that targets women aged 50 years and older. To our knowledge, there currently exists no other programme that specifically targets physical activity information, resources and support in a way that is relevant to the needs of women aged 50 years and over who are insufficiently physically active.

This innovative programme takes a tailored and supported approach to the provision of physical activity information and follow-up support through health coaching and email or SMS messaging and has the potential to substantially increase physical activity participation in women people aged 50 years and over. End user input has been used to design the website to provide relevance and familiarity to the target audience in the delivery of information, resources, inspirational stories and suggestions. Participants are regularly referred to the website via email or SMS messaging and provided with health coaching to further tailor and support their uptake of regular physical activity.

The *Active Women over 50* programme also allows for the targeting of key messages to a broad range of women aged 50 years and over from different geographical areas and sociodemographic backgrounds. If found to be feasible and acceptable in this pilot RCT and effective in a future planned, rigorously conducted RCT, the *Active Women over 50* programme has the potential to be easily scalable and implemented widely to significantly impact the lives of many people.

## Data Availability

Available on request from the corresponding author

## Abbreviations

BCT: Behaviour change technique
CONSORT: CONsolidated Standards Of Reporting Trials
RCT: Randomised controlled trial
REDCap: Research Electronic Data Capture
SDT: Self-determination theory
SMS: Short message service, text messaging
SPIRIT: Standard Protocol Items: Recommendations for Interventional Trials
TIDieR: Template for Intervention Description and Replication
